# Conserved amino acids in the region connecting membrane spanning domain 1 to nucleotide binding domain 1 are essential for expression of the MRP1 (ABCC1) transporter

**DOI:** 10.1371/journal.pone.0246727

**Published:** 2021-02-11

**Authors:** Emma E. Smith, Gwenaëlle Conseil, Susan P. C. Cole

**Affiliations:** 1 Department of Pathology and Molecular Medicine, Queen’s University, Kingston, ON, Canada; 2 Division of Cancer Biology and Genetics, Queen’s University Cancer Research Institute, Kingston, ON, Canada; University of Cambridge, UNITED KINGDOM

## Abstract

Multidrug resistance protein 1 (MRP1) (gene symbol *ABCC1*) is an ATP-binding cassette (ABC) transporter which effluxes xeno- and endobiotic organic anions including estradiol glucuronide and the pro-inflammatory leukotriene C_4_. MRP1 also confers multidrug resistance by reducing intracellular drug accumulation through active efflux. MRP1 has three membrane spanning domains (MSD), and two nucleotide binding domains (NBD). MSD1 and MSD2 are linked to NBD1 and NBD2 by connecting regions (CR) 1 and CR2, respectively. Here we targeted four residues in CR1 (Ser^612^, Arg^615^, His^622^, Glu^624^) for alanine substitution and unexpectedly, found that cellular levels of three mutants (S612A, R615A, E624A) in transfected HEK cells were substantially lower than wild-type MRP1. Whereas CR1-H622A properly trafficked to the plasma membrane and exhibited organic anion transport activity comparable to wild-type MRP1, the poorly expressing R615A and E624A (and to a lesser extent S612A) mutant proteins were retained intracellularly. Analyses of cryogenic electron microscopic and atomic homology models of MRP1 indicated that Arg^615^ and Glu^624^ might participate in bonding interactions with nearby residues to stabilize expression of the transporter. However, this was not supported by double exchange mutations E624K/K406E, R615D/D430R and R615F/F619R which failed to improve MRP1 levels. Nevertheless, these experiments revealed that the highly conserved CR1-Phe^619^ and distal Lys^406^ in the first cytoplasmic loop of MSD1 are also essential for expression of MRP1 protein. This study is the first to demonstrate that CR1 contains several highly conserved residues critical for plasma membrane expression of MRP1 but thus far, currently available structures and models do not provide any insights into the underlying mechanism(s). Additional structures with rigorous biochemical validation data are needed to fully understand the bonding interactions critical to stable expression of this clinically important ABC transporter.

## Introduction

The ATP-binding cassette (ABC) multidrug resistance protein 1 (MRP1) (encoded by *ABCC1*) is expressed at varying levels in most tissues where it effluxes a wide variety of physiologic and xenobiotic organic anions [1–3]. Physiological solutes transported by MRP1 in an ATP-dependent manner include the conjugated steroids estradiol glucuronide (E_2_17βG) and estrone sulphate. In addition, the pro-inflammatory and bronchoconstrictive glutathione (GSH) conjugated cysteinyl leukotriene C_4_ (LTC_4_) is an important physiologic substrate [2, 4–6].

In addition to LTC_4_ and other conjugated organic anions, MRP1 transports amphipathic natural product antineoplastic agents including anthracyclines and *Vinca* alkaloids. It confers resistance to these drugs by reducing their cellular accumulation [7]. Although it plays a lesser role than the ABC drug transporting P-glycoprotein (ABCB1) and ABCG2, MRP1-mediated drug efflux is believed to be clinically relevant in some multidrug resistant tumors and in the tissue distribution of various therapeutic agents [8, 9]. Distinct from P-glycoprotein and ABCG2, however, MRP1-mediated export of natural product drugs and some other xenobiotics and organic anions requires the presence of the physiological tripeptide antioxidant GSH (γ-Glu-Cys-Gly) [10–15]. GSH and its oxidized dimer glutathione disulfide (GSSG) are themselves transported by MRP1 [16–21], suggesting potential roles for MRP1 in a wide variety of GSH/GSSG dependent cellular processes including enzyme function, signal transduction, apoptosis, ferroptosis, protein biosynthesis and assembly as well as neutralization of nitric oxide and reactive oxygen species [22–30].

**Fig 1:**
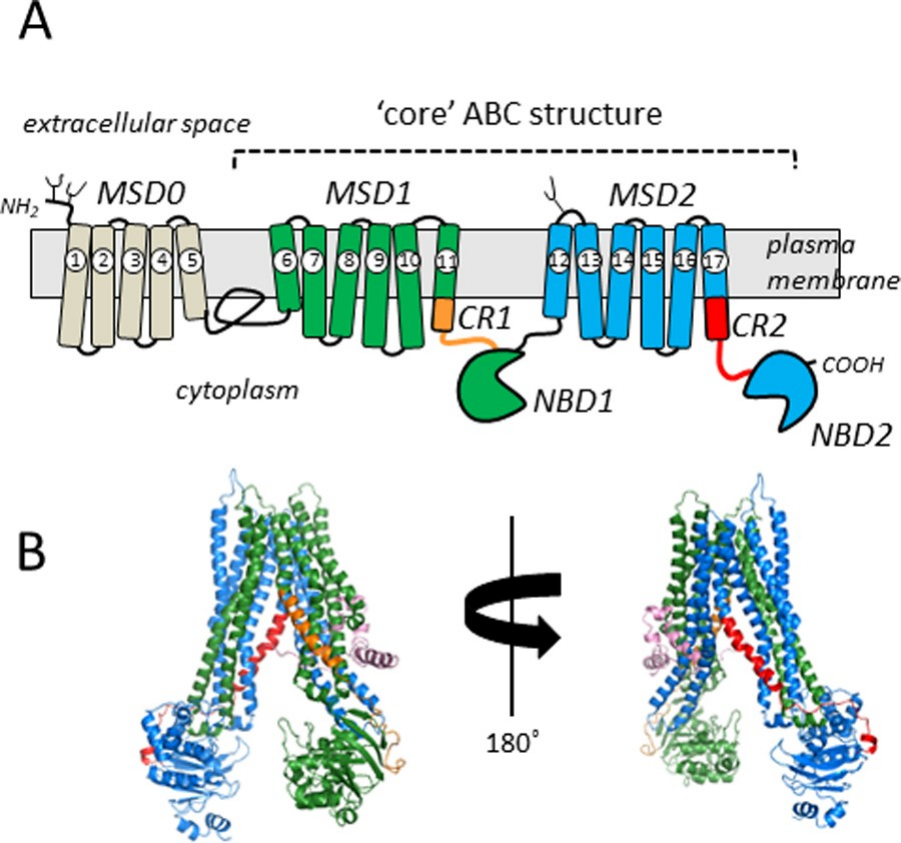
Models of human MRP1. (A) Shown is a two-dimensional illustration of the domain structure of MRP1, a so-called ‘long’ ABCC transporter [58]. MSD0 (*beige*) is present only in a subset of ABCC/MRP subfamily members and is connected to MSD1 by cytoplasmic loop 3 (often referred to as L_o_). The core ABC domain structure is comprised of MSD1/NBD1 (*green*), CR1 (*orange*), MSD2/NBD2 (*blue*) and CR2 (*red*). The TMs of MRP1 are numbered 1–17, and the three N-glycosylation sites at Asn^19^, Asn^23^ and Asn^1006^ are indicated by small branches. (B) Shown are views of a homology model of the core human MRP1 structure [43] based on the cryo-EM structure of apo bovine Mrp1 (PDB ID: 5UJ9) at 0° and 180° rotation on a vertical axis so that CR1 (*orange*) and CR2 (*red*) are clearly visible. The colours of the other domains are the same as in panel (A) with a portion of cytoplasmic loop 3 between MSD0 and MSD1 coloured *pink*. MSD, membrane spanning domain; NBD, nucleotide binding domain; CR, connecting region.

MRP1 has a 5-domain structure comprised of a NH_2_-proximal membrane spanning domain (MSD0) that precedes a ‘core’ structure of MSD1 and MSD2 (each containing six transmembrane (TM) α-helices), each of which is connected to a nucleotide binding domain (NBD1 and NBD2) by stretches of 40–50 amino acids we have termed the ‘connecting regions’ (CR1 and CR2) of the transporter. Thus, the 190 kDa phosphoglycoprotein MRP1 has a modular structure configured MSD0-MSD1-CR1-NBD1-MSD2-CR2-NBD2 (Fig 1A).

MRP1 contains at least four pharmacologically distinct substrate (inhibitor) binding sites with some structural overlap that is not yet precisely defined [31–34]. Nevertheless, there is a considerable body of both functional and structural evidence that supports the view that the TM helices that comprise MSD1 and MSD2 contain most if not all of its substrate binding sites as well as forming the translocation pathway through which solutes are effluxed. In contrast to P-glycoprotein whose multidrug substrates gain access to its efflux pathway through the plasma membrane [35–38], MRP1’s organic anion substrates gain access to their binding sites from the cytoplasm. This is not only because of their (mostly) greater hydrophilicity but also because most of them, including LTC_4_, are formed in intracellular compartments through the action of conjugating enzymes [39].

**Fig 2:**
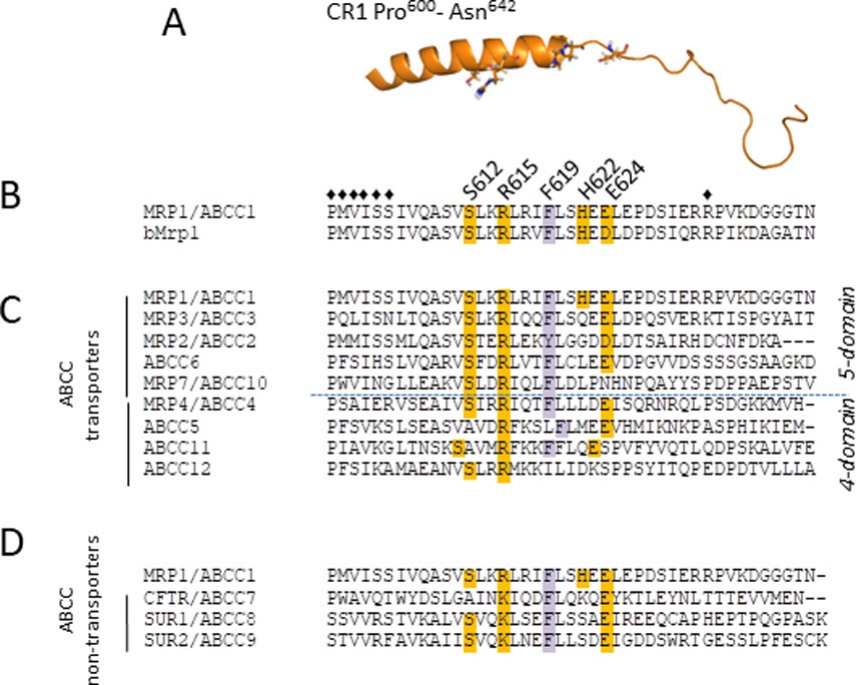
Predicted location and sequence alignments of targeted CR1-MRP1 residues. (A) Shown is a cartoon of the secondary structure of MRP1-CR1 (amino acids 600–642) obtained from the atomic homology model of human MRP1 based on the cryo-EM structure of apo bovine Mrp1 (PDB: 5UJ9) [43]. The side chains of the initial four targeted CR1 residues [Ser^612^, Arg^615^, His^622^, Glu^624^] are shown in stick form and coloured by element where oxygen is *red* and nitrogen is *blue*. Sequence alignments of human MRP1-CR1 with (B) bovine Mrp1; (C) human ABCC 5-domain and 4-domain transporter homologs; and (D) human ABCC non-transporter homologs were generated using Clustal Omega [60]. The initial four and subsequent single CR1 amino acids targeted for mutagenesis in this study (Ser^612^, Arg^615^, His^622^, Glu^624^, and Phe^619^, respectively) are highlighted in *dark orange* and *purple*, respectively. In (B), the diamond symbols (♦) above the sequence denotes the seven amino acids in this region (and the COOH-proximal end of TM11) investigated previously (Pro^600^, Met^601^, Val^602^, Ile^603^, Ser^604^, Ser^605^, Arg^633^) [57, 72, 73] (Cole laboratory, unpublished).

Aided in part by various structures and models [40–43], studies of the substrate selectivity and transport mechanism of MRP1 thus far have mostly focused on investigating its TMs, the cytoplasmic loops (CLs) linking them, and its two functionally asymmetric NBDs [34, 42–50]. In contrast, CR1 and CR2 have been largely neglected (Fig 1; S1 Fig). Informed by *in silico* analyses, we have defined CR1 as Pro^600^-Asn^642^ (Fig 2A). Although there are some differences in the details, all predictive algorithms and homology models of human MRP1 as well as cryoelectrogenic microscopy (cryo-EM) structures of bovine Mrp1 indicate that the NH_2_-proximal half of CR1 is an α-helical extension of TM11 whereas the COOH-proximal half of this ~40 amino acid segment that leads into the β-strand that begins to define NBD1 is intrinsically unstructured [41–43, 51] (Fig 2A). Because CR1 physically links MSD1 to NBD1, we postulated that it could play a role in imparting information about nucleotide occupancy of the NBDs and the substrate binding elements of the MSDs. We reasoned that the unstructured COOH-proximal portion of CR1 may enable it to act as a flexible ‘hinge’ that facilitates conformational changes that occur during substrate binding and/or the transport cycle of MRP1.

To test the hypothesis that CR1 plays a role in MRP1 function, we used primary sequence alignments and available cryo-EM structures and human MRP1 models to select residues in CR1 for mutagenesis. Rather than affecting function, we found that mutation of several conserved CR1 residues resulted in greatly reduced levels of the transporter when expressed in HEK cells.

## Materials and methods

### Site-directed mutagenesis

DNA primers used for mutagenesis were obtained from Integrated DNA Technologies (Coralville, IA) and their sequences are listed in S1 Table. Single MRP1 mutations K406E, S612A, R615A, R615K, F619Y, H622A, and the double exchange mutations K406E/E624K were introduced by a ‘one step’ polymerase chain reaction (PCR) mutagenesis technique using a Quikchange II XL Kit (Agilent Technologies 200521–5, Santa Clara, CA) using full-length wild-type pcDNA3.1(-)-MRP1 and -E624K as templates [43]. All other single and double mutants (K406A, K406R, D430R, D430R/R615D, R615D, R615F, R615F/F619R, F619A, F619R, E624A, E624D, E624K) were generated using a ‘two step’ PCR process using pGEM3Z BamHI/SphI (*ABCC1* mRNA nt 841–2700) and pGEM-3Z-HindIII (*ABCC1* mRNA nt 1547–2875) plasmids as mutagenesis templates followed by subcloning of the mutant restriction fragments into full-length pcDNA3.1(-)-MRP1 [52]. All mutant constructs were fully sequenced (The Center for Applied Genomics, Toronto, ON, Canada) to confirm the presence of the desired mutations and the fidelity of all constructs.

### Cell culture, transfections, and preparation of whole cell extracts and membrane vesicles

SV40-transformed HEK cells were grown in DMEM supplemented with 7.5% fetal bovine serum at 37°C in 5% CO_2_/95% air and transfected with wild-type and mutant pcDNA3.1(-)-MRP1 expression vectors using Lipofectamine^®^ 2000 (Invitrogen 11668–019) (DNA:lipofectamine ratio 1:3) as before [43]. Untransfected cells were included as negative controls. Cells were collected by centrifugation and snap frozen 48 h post-transfection and stored at -80°C until needed. Extracts were prepared by first resuspending the transfected cells in solubilization buffer [10 mM Tris (pH 7.5), 150 mM NaCl, 1 mM EDTA, 0.5% sodium deoxycholate, 0.1% SDS, 1% Triton X-100] with EDTA-free protease inhibitor cocktail (Roche 11836170001) and DNaseI (50 mg ml^-1^) (Amersham Biosciences) and incubating on ice for 10–15 min. The suspension was then centrifuged and protein in the supernatant (referred to hereafter as whole cell extracts; WCE) was quantified using the Bio-Rad *D*_*C*_ Protein Assay with bovine serum albumin (BSA) as a standard. Membrane vesicles were prepared by disrupting slowly thawed transfected cells by argon cavitation (250 psi, 4°C, 5 min), followed by a low speed centrifugation. The supernatant was centrifuged (100,000 xg, 1 h, 4°C) on a 35% sucrose cushion and the membranes at the interface collected and centrifuged again (128,000 xg, 20 min, 4°C). After resuspending the pellet in 50 mM Tris (pH 7.4) and 250 mM sucrose, vesicles were formed by repeated passage through a 27-gauge needle as before [10, 43, 53]. Vesicles were aliquoted and stored at -80°C until needed. Vesicular protein concentrations were determined using the Bradford method with BSA as a standard.

### Immunoblotting

Relative levels of wild-type and mutant MRP1 in WCE and membrane vesicles were determined by immunoblot analysis as before [54] using mouse mAb QCRL-1 anti-human MRP1 (diluted 1:10,000) (epitope amino acids ^918^SSYSGDI^924^ [54]) and mouse anti-α-tubulin (diluted 1:10,000) (Sigma T6074) or rabbit anti-Na^+^/K^+^-ATPase (diluted 1:2,500) (Santa Cruz Biotechnology 28800) as a protein loading control followed by incubation with horseradish peroxidase-conjugated goat anti-mouse (ThermoFisher Scientific 31430) or anti-rabbit (Cedarlane CLAS10-667) antibodies (diluted 1:10,000), respectively. Antibodies bound to the blot were detected using a Western Blotting Chemiluminescence Reagent Plus Kit (PerkinElmer NEL105) and the blot exposed to HyBlot CL autoradiography film (Denville Scientific) for several time periods. Signals on the film were quantified by densitometry using ImageJ software [55] and values adjusted if needed, according to the signal of the protein loading control. Mutant MRP1 levels were then expressed relative to wild-type MRP1 levels and data analysed for differences using GraphPad Prism^TM^ (GraphPad Software, La Jolla, CA) using an unpaired t-test with a significance threshold of *P*<0.05.

### Indirect immunofluorescence confocal microscopy

MRP1 was visualized in intact cells as previously described [53]. Briefly, MRP1 expression vectors were transfected into HEK cells attached to coverslips coated with 0.01% poly-L lysine. After 24 h, transfected cells were fixed (95% ethanol), permeabilized (0.2% Triton X-100/PBS), and blocked (1% BSA/0.1% Triton X-100/PBS) before staining with anti-MRP1 rat mAb MRPr1 (epitope amino acids ^238^GSDLWSLNKE^247^ [56]) (diluted 1:1,000) (gift of Dr. R. Scheper) followed by incubation with secondary Alexa Fluor^TM^ 488 conjugated goat anti-rat antibody (diluted 1:300) (Invitrogen A11006); nuclei were stained with 4′,6-diamidino-2-phenylindole (Sigma Aldrich) (diluted 1:10,000). Coverslips were mounted face down on glass slides on a drop of Slow Fade^®^ Gold antifade reagent (Life Technologies). Images were obtained at the Queen’s University Biomedical Imaging Center using a spinning Quorum Wave Effects Spinning disk confocal microscope. MetaMorph^®^ microscopy automation and imaging analysis software (Molecular Devices) were used to analyse the captured images.

### Vesicular transport assays

ATP-dependent transport of ^3^H-labeled organic anions by wild-type and mutant MRP1 was determined using a vesicular transport assay adapted to a 96-well plate format, performed in duplicate, as previously described [10, 43, 57]. Briefly, each assay tube contained 10 mM MgCl_2_, either 4 mM ATP or AMP together with unlabeled and tritiated forms of organic anion substrate according to the following conditions pre-determined to ensure that transport was linear up until the time indicated: for LTC_4_ uptake, 50 nM LTC_4_ (Cedarlane), 10 nCi [^3^H]LTC_4_ (170.2 Ci mmol^-1^) (Perkin Elmer NET1018), 2 μg vesicle protein, 4 min at 23°C; and for E_2_17βG uptake, 400 nM E_2_17βG (Sigma Aldrich), 20 nCi [^3^H]E_2_17βG (52.9 Ci mmol^-1^) (Perkin Elmer NET1106), 2 μg vesicle protein, 5 min at 37°C. Uptake into the inside-out vesicles was stopped by diluting the assay mixture in ice-cold Tris-sucrose buffer and the vesicles captured by filtering through a Unifilter GF/B plate using a Packard Filtermate Harvester. After drying overnight, Microscint^TM^ (Perkin Elmer) was added to the filter plate and tritium quantified. ATP-dependent uptake of organic anions into the inside-out vesicles was calculated by subtracting uptake of ^3^H-labeled substrate by MRP1-enriched vesicles in the presence of AMP from uptake in the presence of ATP. If needed, transport levels were adjusted to take into account the differences in levels of wild-type and mutant MRP1 in the vesicles as determined by prior immunoblotting, and then mutant MRP1 transport activity was expressed as a percent of wild-type MRP1 activity. When three or more experiments were completed, an unpaired t-test was performed using GraphPad Prism to determine whether or not differences were significant with a threshold of *P*<0.05.

## Results

### Sequence alignments and initial selection of CR1 amino acids for targeted mutagenesis

To determine which CR1 residues to target for alanine substitution, multiple sequence alignments of MRP1 CR1 (amino acids 600–642) with the comparable region of bovine Mrp1 (used in cryo-EM studies), the eight other transporting ABCC subfamily members [58, 59], as well as the three non-transporting ABCC subfamily members were carried out using Clustal Omega [60] (Fig 2B–2D). As expected, conservation between human and bovine MRP1/Mrp1 is very high with 36 of 43 amino acids being identical (Fig 2B). There is also substantial conservation among human ABCC homologs particularly with the other 5-domain transporters MRP3/ABCC3, MRP2/ABCC2 and ABCC6 (Fig 2C). On the other hand, MRP1-CR1 is less well conserved in the three non-transporting human ABCC homologs which include the cystic fibrosis transmembrane conductance regulator CFTR/ABCC7, and the two sulfonylurea receptors SUR1/ABCC8 and SUR2/ABCC9 (Fig 2D). In general, the NH_2_-proximal α-helical half of CR1 shows a higher degree of conservation than the unstructured COOH-proximal half. Based on these alignments, CR1 residues Ser^612^, Arg^615^ and Glu^624^ were targeted for mutagenesis not only because of their almost invariant occurrence in ABCC subfamily members but also because they contain polar side chains with potential for involvement in interatomic bonding interactions. A fourth CR1 amino acid, His^622^, was targeted because of its predicted location at the potentially important junction of the α-helical and unstructured regions of CR1.

### Effect of Ala substitution of CR1 residues Ser^612^, Arg^615^, His^622^ and Glu^624^ on MRP1 levels and plasma membrane localization

**Fig 3:**
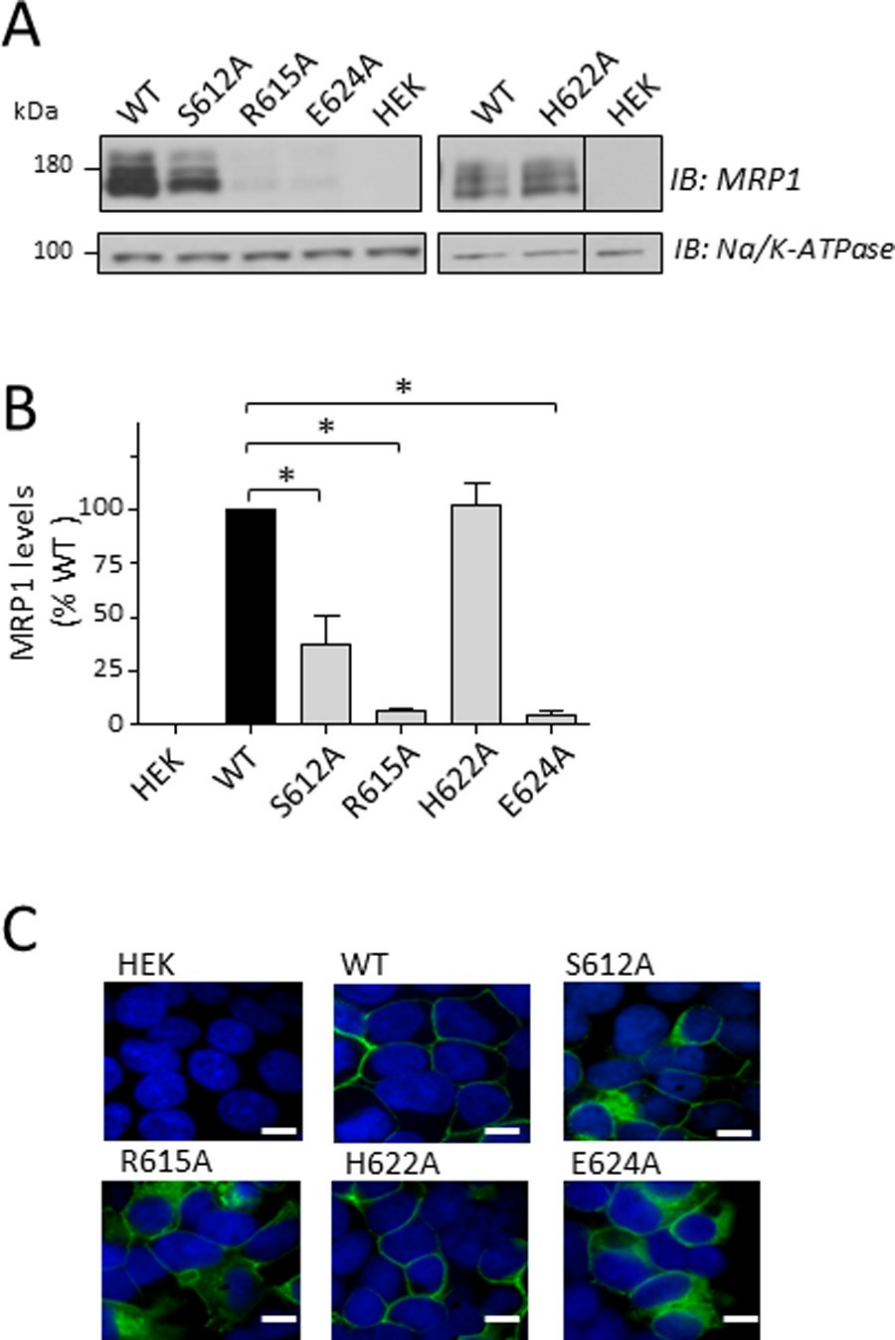
CR1 mutations S612A, R615A, H622A and E624A have different effects on total and membrane MRP1 levels in transfected HEK cells. (A) Shown are representative immunoblots of whole cell extracts (10 μg protein loaded per lane) prepared from HEK cells transfected with wild-type (WT) and mutant (S612A, R615A, H622A, E624A) MRP1 pcDNA expression vectors as well as untransfected cells (HEK) (negative control). MRP1 was detected with mAb QCRL-1 [54], and anti-Na^+^/K^+^-ATPase served as a protein loading control. A vertical black line indicates where irrelevant lanes from the immunoblot were removed by cropping images; the vertical space between lanes indicates independent immunoblots. The images in each panel are from a single blot with the region between MRP1 and the loading control cropped out. (B) WT and S612A, R615A, H622A and E624A mutant MRP1 levels, normalized for protein loading based on the Na^+^/K^+^-ATPase signal, were estimated using densitometry and plotted as a percent of wild-type MRP1 levels. Bars represent the mean values (± SD) of results obtained from three independent transfections. * *P*<0.05, unpaired t-test. (C) Shown are non-quantitative confocal microscopy images of HEK cells 24 h after transfection with WT and CR1 mutant (S612A, R615A, H622A, E624A) pcDNA expression vectors. MRP1 (*green*) was detected using rat mAb MRPr1 [56] and Alexa Fluor 488-conjugated goat anti-rat as the primary and secondary antibodies, respectively. Untransfected cells (HEK) were used as a negative control. Nuclei are stained *blue*. Signals from the two channels were acquired independently and the merged images are presented. White calibration bars (*bottom right*) represent 10 μm.

Alanine substitutions of Ser^612^, Arg^615^, His^622^ and Glu^624^ were generated by site-directed mutagenesis, and the mutant MRP1 constructs transfected into HEK cells. After 48 h, cells were collected, extracts prepared from frozen cell pellets and total cellular MRP1 levels estimated by immunoblotting and densitometry. As shown in Fig 3A and 3B, two of four CR1 mutant proteins were barely detectable (R615A, E624A) (≤6% wild-type MRP1; *P*<0.001) and levels of the S612A mutant were decreased by 63 ± 14% (*P* = 0.0015). In contrast, H622A levels were comparable to wild-type MRP1 (102 ± 11%; *P* = 0.7931). To ensure that alanine substitution of the targeted CR1 residues was the sole reason for the reduced levels of the S612A, R615A and E624A mutants, the mutant expression constructs were mutated back to wild-type MRP1 and once again expressed in HEK cells. In all cases, the revertants were expressed at levels comparable to wild-type MRP1 (results not shown). These observations indicate that Arg^615^ and Glu^624^, and to a lesser extent Ser^612^, but not His^622^, contribute to stable expression of MRP1 in HEK cells.

To investigate whether the alanine substitutions of CR1 residues Ser^612^, Arg^615^, His^622^ or Glu^624^ affected the ability of MRP1 to traffic to the plasma membrane, the mutant proteins were visualized in intact HEK cells 24 h after transfection using indirect immunofluorescent confocal microscopy. As shown in Fig 3C, the signal corresponding to MRP1 was consistent with a plasma membrane localization in cells transfected with the abundantly expressed wild-type and H622A mutant MRP1. In contrast, the small amounts of detectable R615A and E624A proteins were retained intracellularly; some S612A mutant protein was found at the plasma membrane but a substantial proportion of it was retained intracellularly. These results are consistent with the immunoblots of WCEs where the Ala-substituted mutants with the most severely disrupted cellular localization (R615A, E624A) were detected at significantly lower levels than the moderately disrupted S612A (Fig 3A).

### MRP1 transport activity is not affected by the H622A mutation

**Fig 4:**
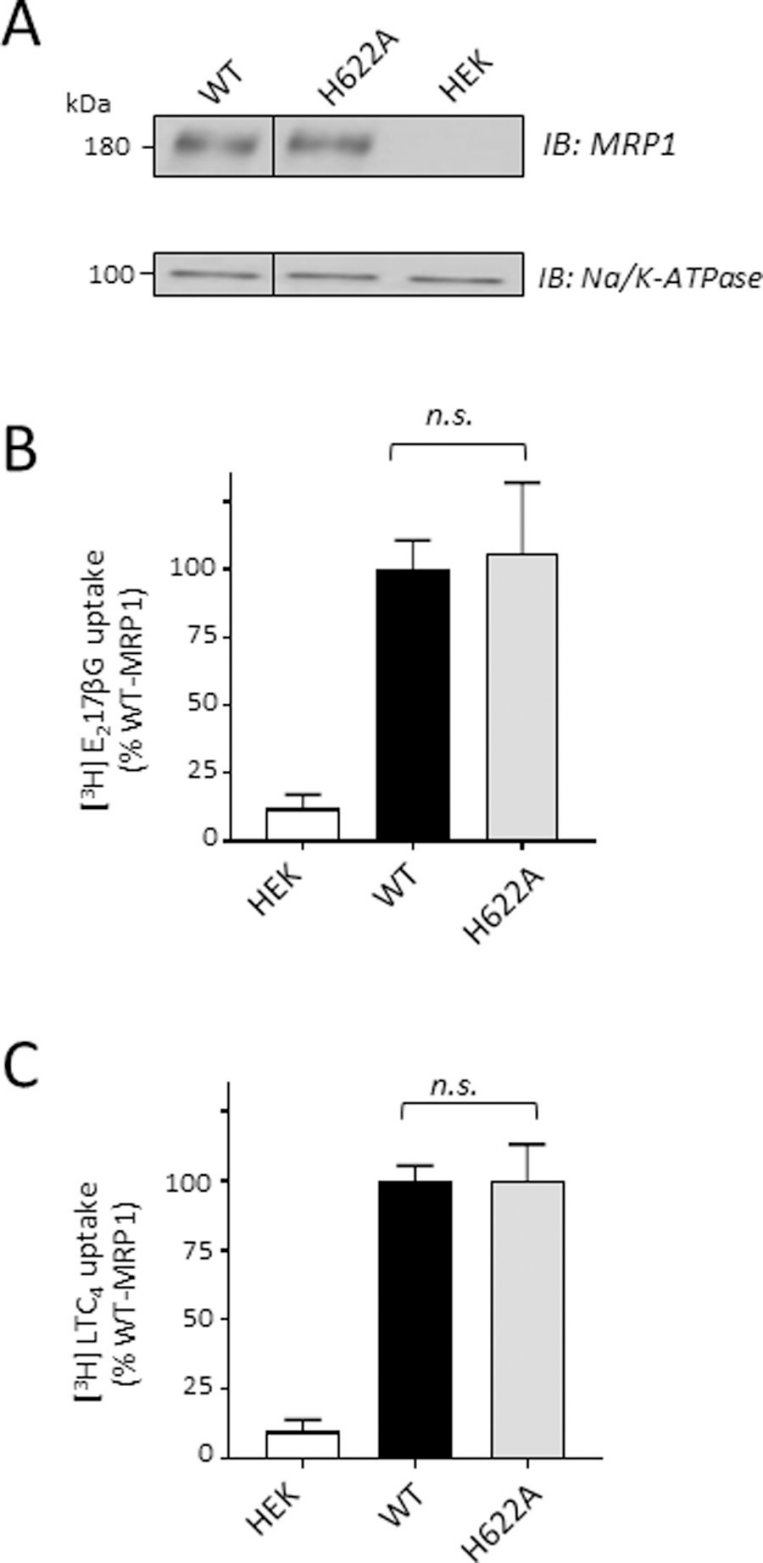
Ala substitution of CR1-His^622^ has no effect on levels or organic anion transport activity of MRP1. (A) Shown is a representative immunoblot of inside-out membrane vesicles (1 μg protein per lane) prepared from HEK cells transfected with wild-type (WT) and H622A MRP1 pcDNA expression vectors as well as untransfected cells (HEK) (negative control). The blot was probed for MRP1 (Mab QCRL-1) [54] and Na^+^/K^+^-ATPase as a loading control. The vertical black line indicates where irrelevant lanes from the immunoblot were removed by cropping the image. The images in each panel are from a single blot with the region between MRP1 and the loading control (Na^+^/K^+^-ATPase*)* cropped out. (B & C) Transport activity was measured as ATP-dependent uptake of (B) [^3^H]E_2_17βG and (C) [^3^H]LTC_4_ into inside-out membrane vesicles and expressed as a percent of wild-type MRP1 uptake. The values shown have been adjusted to take into account minor differences in mutant MRP1 levels in the membrane vesicles relative to wild-type MRP1. Bars represent the mean values (± SD) of results obtained from three independent experiments. *n*.*s*., not significant *P*>0.05, unpaired t-test.

Given that H622A levels and membrane localization were comparable to wild-type MRP1, it was of interest to determine if this mutation affected the transport activity of MRP1. Accordingly, membrane vesicles were prepared from HEK cells expressing MRP1-H622A and immunoblotting showed that vesicular levels of H622A were comparable to wild-type MRP1 (Fig 4A) as observed for WCE (Fig 3A). Subsequent transport assays showed that levels of ATP-dependent [^3^H]E_2_17βG and [^3^H]LTC_4_ uptake into inside-out membrane vesicles were also unchanged (106 ± 26% and 100 ± 13% of wild-type MRP1 levels, respectively; *P* = 0.7307 and *P*>0.9999, respectively) (Fig 4B and 4C). Taken together, these data indicate that the non-conservative substitution of CR1-His^622^ with a cavity-creating chargeless alanine has no deleterious effect on either the levels or function of MRP1.

### *In silico* analyses suggest mutation-sensitive CR1 Arg^615^ and Glu^624^ participate in stabilizing bonding interactions with nearby amino acids

The profound destabilizing effect of replacing either CR1 Arg^615^ or Glu^624^ with alanine was unexpected because residues within CR1 have never previously been implicated as playing a role in stable expression of MRP1 or other MRP transporter. For this reason, using the ‘measurement’ tool of the molecular visualization program PyMol, we examined the cryo-EM structure of apo bovine Mrp1 [41], as well as atomic homology models of apo human MRP1 based on this structure [43] and the crystal structure of apo TM287/288 from *T*. *maritima* [42] to determine if they might suggest the existence of electrostatic bonding interactions that could help explain the reduced levels of the R615A and E624A mutants.

**Fig 5:**
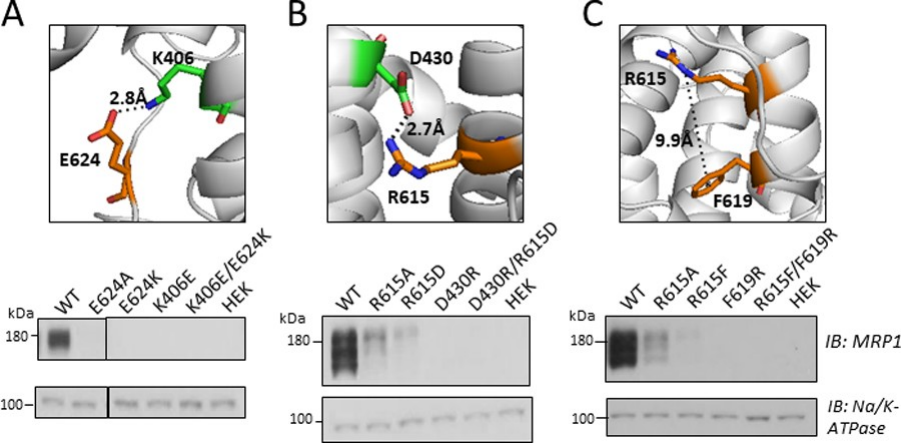
Potential bonding interactions of CR1 Arg^615^ and Glu^624^ and effect of structure-guided double exchange mutations on MRP1 levels. *Upper panels*, Shown are potential electrostatic bonding interactions between (A) Lys^406^ and Glu^624^; and (B) Arg^615^ and Asp^430^, and (C) Arg^615^ and Phe^619^ derived from an atomic homology model of human MRP1 [43] based on the apo bovine Mrp1 cryo-EM structure (PDB ID: 5UJ9). While several bonding interactions are theoretically possible, for clarity, only the potential bond (*dotted line*) with the shortest predicted distance between each pair of amino acids is shown. Side chains are shown as sticks and coloured by element where oxygen is *red* and nitrogen is *blu*e. *Lower panels*, Shown are representative immunoblots of cell extracts (10 μg protein per lane) prepared from HEK cells transfected with (A) wild-type (WT) and mutant (E624A, E624K, K406E, K406E/E624K) MRP1 and (B) WT and mutant (R615A, R615D, D430R, D430R/R615D) MRP1; and (C) WT and mutant (R615A, R615F, F619R, R615F/F619R) pcDNA expression vectors. Extracts from untransfected cells (HEK) served as negative controls. MRP1 was detected with mAb QCRL-1 [54], and anti-Na^+^/K^+^-ATPase was used as a protein loading control. The images in each panel are from a single blot with the region between MRP1 and the loading control cropped out. The vertical black lines in (A) indicate where irrelevant lanes from the immunoblot were removed by cropping images. Similar results were obtained with extracts from two independent transfections.

Possible stabilizing salt-bridges (ionic interactions) that could occur between Arg^615^ and Glu^624^ and nearby amino acids were considered possible if the charged atom of the side chain of either of these two residues was within 5Å of an opposite charged atom on the side chain of another residue [61, 62]. In addition, an electrostatic cation-π interaction was considered possible if the cationic guanidinium group of Arg^615^ was within 6.6Å of the π electron system of an amino acid with an aromatic center (Phe, Trp, Tyr) [63, 64]. These analyses revealed interatomic distances in the static cryo-EM structure of apo bovine Mrp1 [41] and the two human apo MRP1 homology models [42, 43] between the two oxygen atoms of the CR1-Glu^624^ γ-carboxylate group and the cationic ε-amino group of Lys^406^ in CL4 ranging from 2.7Å to 5.7Å, supporting the possibility of a salt-bridge between these residues (Fig 5A, *upper panel*). The models also show multiple potential salt-bridges between the atoms of the cationic guanidinium group of CR1-Arg^615^ and the two oxygen atoms of the β-carboxylate group of CL4-Asp^430^ at the juxtamembrane region of TM8, with interatomic distances ranging from 2.7Å to 4.8Å in both human MRP1 homology models depending on the participating atoms (Fig 5B, *upper panel*).

Lastly, our *in silico* analyses indicated the relatively close proximity of CR1-Arg^615^ to CR1-Phe^619^ (Fig 5C, *upper panel*). Although the distances between the positive atoms of the Arg^615^ guanidinium group and the aromatic center of Phe^619^ were estimated to be at least 9Å (a distance substantially greater than the 6.6Å considered maximal for a cation-π interaction to occur [64]), it is noted that the apo bovine Mrp1 cryo-EM structure was solved at a resolution of just 3.49Å [41] and thus it is possible that the actual distance between the side chains in this dynamic region could be less than indicated by the static structures and models [65]. For this reason, the potential existence of an intrahelical cation-π bond between CR1-Arg^615^ and CR1-Phe^619^ was also investigated.

### Effect of CR1 exchange mutations of Arg^615^ and Glu^624^ on MRP1 levels in HEK cells

We reasoned that if the loss of stabilizing salt-bridges contributes to the low levels of R615A and E624A, then reciprocal charge exchange mutations of Arg^615^ or Glu^624^ that are expected to preserve the electrostatic bonds might restore or at least improve MRP1 expression levels as has been shown in several other polytopic membrane proteins [66–69]. To explore this possibility for CR1-Glu^624^, the double charge exchange mutant K406E/E624K and corresponding single mutant controls E624K and K406E were made and their levels in extracts prepared from transfected cells determined by immunoblotting. These experiments showed that both the single E624K and K406E mutants as well as the double exchange mutant K406E/E624K were expressed very poorly (levels <5% wild-type MRP1) and not detectable in immunoblots after a typical film exposure time (10 s) (Fig 5A, *lower panel*). After a prolonged exposure (1 min), a small amount of underglycosylated E624K was visible but K406E and K406E/E624K remained undetectable (not shown). These observations indicate that rather than improving MRP1 levels as anticipated, the double charge exchange mutations K406E/E624K diminished them. On the other hand, these experiments revealed for the first time that Lys^406^ in the CL (CL4) linking TM7 to TM8 is another potentially mutation-sensitive amino acid involved in stable expression of MRP1.

Possible salt-bridges between CR1-Arg^615^ and CL4/TM8-Asp^430^ (Fig 5B, *upper panel*) were also investigated by creating D430R and R615D single controls and D430R/R615D double charge exchange mutant constructs, transfecting them into HEK cells and extracts prepared and immunoblotted as before. We have previously reported that the MRP1 mutant D430R is not expressed [70] and this was confirmed here, as shown in Fig 5B *(lower panel)*. Indeed, D430R as well as R615D, and D430R/R615D were all expressed at levels that were <5% of wild-type MRP1. After a typical (10 s) film exposure, a faint signal corresponding to mature, glycosylated R615D was visible, but not for D430R or D430R/R615D. After a prolonged exposure (1 min), faint signals corresponding to underglycosylated D430R and glycosylated D430R/R615D were detected although still substantially lower than wild-type MRP1 and the single R615A and R615D mutants (not shown). The inability of charge exchange mutagenesis of CR1-Arg^615^ with CL4/TM8-Asp^430^ to improve MRP1 levels does not support the presence of a stabilizing salt bridge between these two residues.

Lastly, a possible stabilizing cation-π interaction within CR1 between Arg^615^ and Phe^619^ (Fig 5C, *upper panel*) was investigated by generating single mutant R615F and F619R control and double exchange mutant R615F/F619R constructs, transfecting them in HEK cells and MRP1 levels analysed by immunoblotting of extracts as before. As shown in Fig 5C *(lower panel)*, both the single R615F and F619R mutants as well as the exchange mutant R615F/F619R were expressed at substantially lower levels than both wild-type and R615A MRP1. A typical (10 s) film exposure showed that levels of R615F were <5% wild-type MRP1 levels in WCE, and neither F619R nor R615F/F619R were detectable. After prolonged exposure (1 min), very low levels of an underglycosylated form of F619R were seen but R615F/F619R remained undetectable (not shown). These observations do not support the presence of a stabilizing bonding interaction between Arg^615^ and Phe^619^. On the other hand, they are the first to identify the highly conserved Phe^619^ in the α-helical NH_2_-proximal portion of CR1 as a potentially mutation-sensitive residue essential for stable MRP1 expression.

### Mutational analyses of Lys^406^, Glu^624^, Arg^615^ and Phe^619^

**Fig 6:**
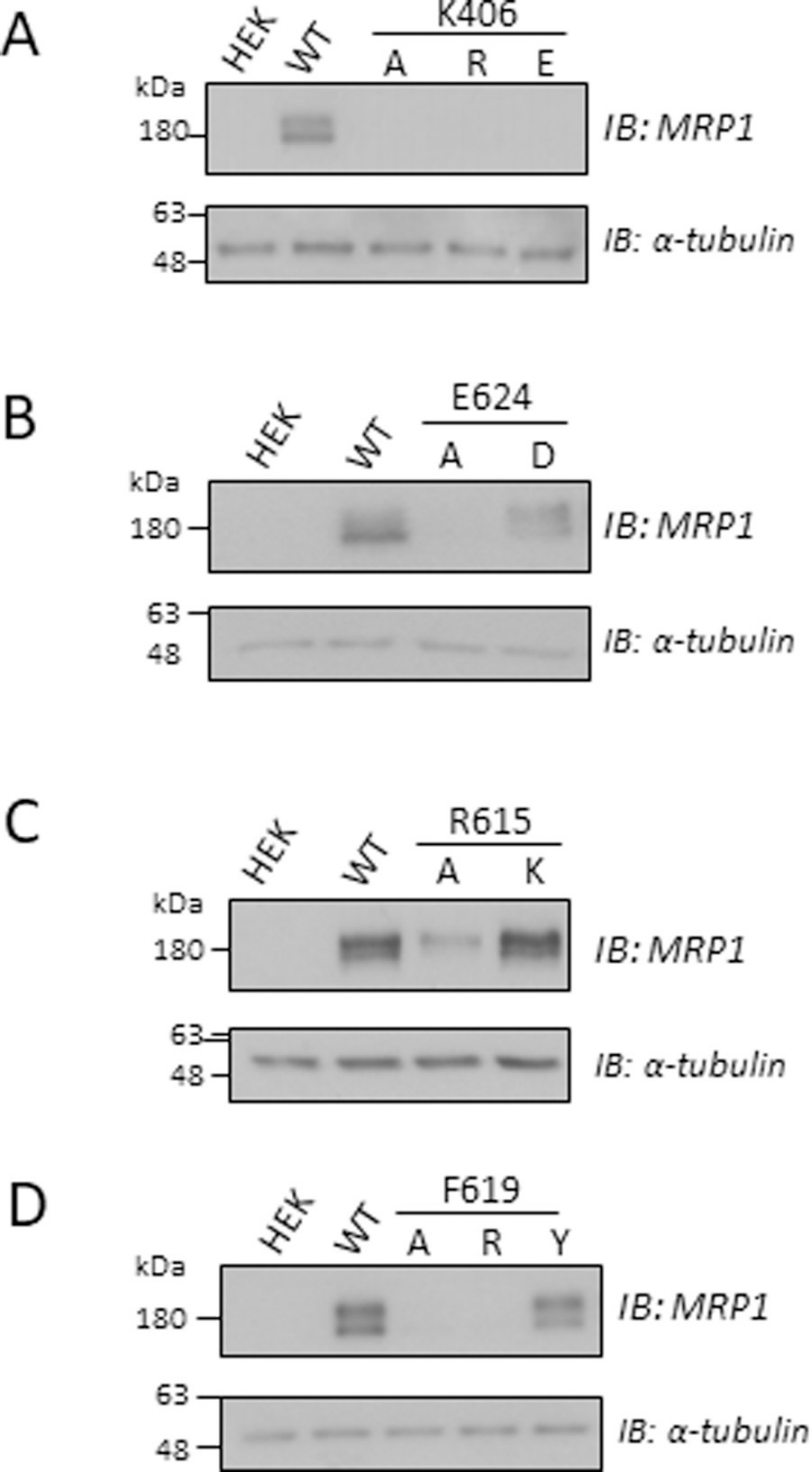
Levels of mutant MRP1 proteins in transfected HEK cells. Shown are immunoblots of cell extracts (10 μg protein per lane) prepared from HEK cells transfected with pcDNA3.1(-) expression vectors encoding wild-type MRP1 (WT) and (A) single K406A/R/E mutants; (B) single E624A/D mutants; (C) single R615A/K mutants; and (D) single F619A/R/Y mutants. Untransfected cells (HEK) served as a negative control. Relative MRP1 levels were quantified by densitometry and adjusted as needed to take into account differences in gel loading by probing blots with antibodies against α-tubulin (loading control). Blots shown are from single experiments; comparable results were obtained in at least one additional independent transfection and immunoblot. For conservative mutants R615K, E624D, and F619Y, levels were comparable to wild-type hMRP1 (1.3, 0.8, and 1.0-fold, respectively; means of 2 independent observations). The images in each panel are from a single blot with the region between MRP1 and the loading control cropped out.

Although the exchange mutagenesis experiments failed to provide any evidence of stabilizing electrostatic interactions of Arg^615^ and Glu^624^ with other nearby residues as predicted by our *in silico* analyses, they unexpectedly revealed both CL4-Lys^406^ and CR1-Phe^619^ to be mutation-sensitive amino acids of MRP1. To explore further what features of these residues are needed for stable MRP1 expression, additional single mutants of CL4-Lys^406^, CR1-Arg^615^, CR1-Phe^619^ as well as CR1-Glu^624^, were generated, expressed in HEK cells and cell extracts probed for MRP1 levels by immunoblotting as before. As shown in Fig 6A, the cavity-creating chargeless K406A and even the same charge K406R mutations resulted in very low MRP1 levels similar to those of the oppositely charged K406E mutant (Fig 5A). In contrast, the same charge mutant of CR1-Glu^624^ (E624D) was expressed at wild-type MRP1 levels (Fig 6B) indicating that despite the shorter length of the aspartate side chain, simply the presence of a negative charge at position 624 in CR1 is sufficient for stable MRP1 expression. This was also the case for CR1-Arg^615^ (Fig 6C) and CL4-Asp^430^ (not shown) where levels of same charge mutants R615K and D430E were comparable to wild-type MRP1. Finally, with respect to CR1-Phe^619^, the cavity-creating neutral F619A mutant was poorly expressed as was the non-conservative F619R; however, levels of the conservatively substituted F619Y mutant were similar to wild-type MRP1 (Fig 6D).

## Discussion

The original objective of this study was to explore the idea that CR1 might play a role in facilitating substrate binding and transport-related conformational changes in MRP1 because of its hinge-like structure and location between MSD1 and NBD1. Accordingly, we replaced four amino acids in CR1 individually with a cavity-creating non-polar alanine and investigated the effects of these non-conservative substitutions on MRP1. Unexpectedly, we found that three of the four Ala-substituted CR1 mutants (S612A, R615A, E624A) were expressed at levels substantially lower than wild-type MRP1; CR1-H622A was the only mutant that exhibited a phenotype comparable to wild-type MRP1. Although poorly conserved among ABCC transporters, His^622^ was originally targeted for mutagenesis because it was postulated that its presumed location at the junction of the α-helical and unstructured halves of CR1 might impart functional importance. However, not only did alanine substitution of CR1-His^622^ not affect MRP1 expression levels, it also had no effect on either the plasma membrane localization of the transporter or its organic anion transport activity. These observations show that despite the location of this positive, bulky, aromatic residue in CR1, His^622^ is not important for either the stable expression or function of MRP1.

**Fig 7:**
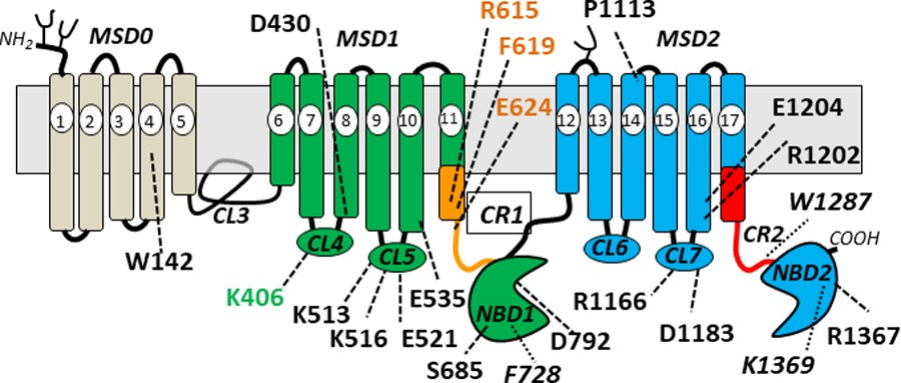
Location of amino acids found essential for stable expression of human MRP1 in mammalian HEK cells. Shown are the locations of amino acids identified in this study and in previously published studies [48, 59, 63, 88] or in unpublished work (Cole laboratory) to be essential for stable MRP1 expression in HEK cells. Amino acids are considered essential if their mutation to a non-conservative or conservative amino acid reduced MRP1 levels by >80% as measured by immunoblotting. Critical amino acids identified in the present study are colored to match their domains (*green*, *orange*). Those from previous studies are uncolored in regular font while those from unpublished work are uncolored in *italics*. MSD, membrane spanning domain; CL, cytoplasmic loop; NBD, nucleotide binding domain; CR, connecting region.

The low levels of the remaining three CR1 mutants indicate that rather than having a role in substrate binding and transport as we originally postulated, these highly conserved polar amino acids all contribute in some way to the stable expression of MRP1. It seems likely they do so by participating in bonding interactions that promote proper intradomain folding and interdomain assembly of the transporter during MRP1 biosynthesis [71]. The extremely low-level CR1 mutants R615A and E624A also both exhibited profoundly disrupted plasma membrane localization with essentially any detectable mutant protein being retained intracellularly whereas a significant portion of the moderately expressed S612A mutant was properly localized to the plasma membrane. These observations suggest that the highly conserved charged Arg^615^ and Glu^624^ in CR1 play a substantially greater role in promoting proper folding and stable plasma membrane expression of MRP1 than the polar Ser^612^. No previous mutations of amino acids located in CR1 or even the COOH-proximal region of TM11 (i.e. Pro^600^, Met^601^, Val^602^, Ile^603^, Ser^604^, Ser^605^, Arg^633^) (Fig 2A) have been found to adversely affect MRP1 levels or disrupt its membrane localization [57, 72, 73; Cole laboratory, unpublished results]. Consequently, this study is the first to provide biochemical evidence that CR1 contains amino acids crucial for stable expression of this transporter. As such, CR1 joins the previously identified cytoplasmic CL5 [50], CL7 [48] and the NBDs [47, 49, 50] as a key region whose integrity is essential for proper folding and plasma membrane localization of MRP1 (Fig 7) [71].

To investigate the possible mechanism underlying the low levels of the most severely disrupted mutants R615A and E624A, we carried out *in silico* analyses of the apo bovine Mrp1 cryo-EM structure as well as two homology models of human MRP1 and identified several potential stabilizing interactions of Arg^615^ and Glu^624^. If the loss of a stabilizing bond causes or contributes to the low levels of their Ala-substituted mutants, it was anticipated that a reciprocal exchange mutant which preserves the putative interaction would increase or restore expression of the mutant MRP1 to wild-type levels. However, this was not the case and the levels of all three exchange mutants tested (E624K/K406E, R615D/D430R, R615F/F619R) remained >80% lower than wild-type MRP1 and comparable to the poorly expressed single Ala-substituted mutants R615K and E624A. Thus, exchange mutagenesis did not provide any supportive evidence of the existence of three potential bonding interactions suggested by the cryo-EM and homology models. These results do not necessarily preclude the existence of these predicted bonding interactions of Arg^615^ and Glu^624^ because it may be that the exchange mutations created a net destabilizing effect on the protein because the targeted residues participate in multiple critical inter- and/or intra-helical bonding interactions which were disrupted by the double mutations. However, another possible explanation is that the orientation of the relevant amino acid side chains in the current static models of MRP1 differ from those that exist under more dynamic physiological conditions. Thus, the models may not reveal the most critical stabilizing bonding interactions in native MRP1. In this regard, it could be relevant that the purified bovine Mrp1 that was used for cryo-EM studies was unglycosylated and purified from sodium butyrate-treated cells incubated at a subphysiological temperature [41]. Future modeling studies using molecular dynamics simulations may be helpful in addressing this possibility [74]. Regardless, it is worth noting that the conservatively substituted CR1 mutants E624D and R615K were expressed at levels comparable to wild-type MRP1, indicating that whatever stabilizing interactions these two amino acids form in native MRP1, they can be maintained simply by preserving the charges of their side chains. In the case of E624D, this is not surprising since one of the very few sequence differences between human MRP1 and its bovine and other Mrp1 orthologs is the presence of an aspartate rather than a glutamate at this position.

Despite the inability of exchange mutagenesis to provide any mechanistic understanding of the destabilizing consequences of mutating CR1 Arg^615^ and Glu^624^, these experiments revealed for the first time a critical role for CR1-Phe^619^ and CL4-Lys^406^ in stable MRP1 expression. In addition, they corroborate our previous report identifying the highly conserved CL4/TM8-Asp^430^ as a similarly mutation-sensitive amino acid [70]. A crucial step in the proper intradomain folding and interdomain assembly of ABC exporters requires the engagement of the second CL of each MSD into a ‘hollow’ in the opposite NBD (in the case of MRP1, MSD1-CL5 and MSD2-CL7 into NBD2 and NBD1, respectively), a process stabilized by charge interactions in conserved positions [71, 75]. Our previous studies demonstrating the importance of charged amino acids in CL5 and CL7 of MRP1 are consistent with this requirement [48, 50]. However, the highly conserved charged Lys^406^ and Asp^430^ are located in the first CL (CL4) of MSD1 and we previously showed that non-conservative substitutions of other charged residues in this loop (i.e. Arg^394^, Lys^396^, Arg^433^, Asp^436^) had no adverse effect on MRP1 levels [70, 76]. While structural studies of ABC transporters indicate that this first CL in MSD1 has a functional role in coupling nucleotide occupancy of NBD1 with the orientation of the TM helices [51, 75], there is a paucity of biochemical evidence supporting a role for this region in promoting stable protein expression. Our observations with Lys^406^ and Asp^430^ mutants indicate that MRP1 could be an exception as is CFTR/ABCC7 where it has been reported that three different mutations in its comparable CL1 (H139R, G149R, D192G) impede its processing resulting in substantially reduced levels of this chloride channel protein [77]. Thus, while formation of a proper interface between the first CL of MSD1 and NBD1 is generally held to be essential for protein function [77–80], for MRP1 (and CFTR), it seems that at least some of the amino acids of CL4 (Lys^406^, Asp^430^) are also needed for proper folding and membrane localization of the transporter. It is of interest that not only is the cavity-creating non-conservative CL4-K406A mutant poorly expressed but so too is the same charge K406R mutant. This indicates that while a positive charge at position 406 is important for MRP1 protein expression, it is not sufficient because the larger volume, distinctive geometry and/or different bonding properties of an arginine side chain cannot be accommodated. In contrast, simply maintaining the charge at position 430 (as in the CL4 mutant D430E) is all that is required for stable expression of MRP1 (results not shown).

Our finding that non-conservative mutations of the highly conserved CR1-Phe^619^ disrupt MRP1 expression was somewhat surprising and is the first time a non-polar aromatic residue has been implicated in stable MRP1 expression. However, the analogous residue in CFTR (Phe^374^) has also been reported to be important for stability of this chloride channel [81]. Our demonstration that the conservatively substituted F619Y mutant could be expressed at wild-type MRP1 levels indicates that the presence of a polar substituent on the amino acid side chain at position 619 does not disrupt MRP1 stability as long as aromaticity is preserved. These observations are consistent with the idea that the CR1-Phe^619^ side chain may participate in stabilizing π-π stacking and/or π-cation interactions with nearby aromatic or positively charged amino acids, respectively [63], at least at some point during MRP1 biosynthesis and/or assembly. However, such interactions are not apparent in any of the current static models of MRP1, and thus how Phe^619^ in MRP1 (and Phe^374^ in CFTR) promotes stable protein expression in mammalian cells remains unknown.

As is frequently the case for poorly expressed ABC proteins [71], the low levels of CR1 mutants R615A, E624A, F619A and CL4 mutants K406A/R and D430A are likely caused by misfolding of MRP1 leading to premature protein degradation by the ubiquitin-proteasome system [82]. Previous studies have shown that misfolded ABC proteins, including MRP1 [83, 84], can sometimes be rescued to varying degrees by proteasome inhibitors or by small molecules acting as so-called pharmacological chaperones [71, 83, 85]. However, in preliminary experiments, exposure of CR1 mutant S612A, R615A, E624A transfected cells to the specific and widely used 20S proteasome inhibitor bortezomib [86, 87] did not substantially increase levels of the fully glycosylated mature 190 kDa form of the mutant MRP1 proteins (S2 Fig). Bortezomib did however increase high molecular weight aggregates of both wild-type and mutant MRP1, but such aggregates are most unlikely to be functional. The inability of bortezomib to restore or even enhance levels of non-aggregated CR1 mutant MRP1 proteins raises the possibility that the mutants are so grossly misfolded that they are overwhelmingly disposed to aggregate. Alternatively, they may be degraded by a proteasome that is resistant to bortezomib although this remains to be investigated [87].

In summary, we have demonstrated for the first time that CR1 which links TM11 of MSD1 to NBD1 contains several amino acids critical for stable expression of MRP1 in mammalian cells and thus this work substantially extends our knowledge of regions and specific amino acids that participate in the proper folding and assembly of this 5-domain organic anion and multidrug ABCC transporter. This in turn may be relevant to other clinically important human ABCC transporters. Disappointingly, however, the mechanism(s) underlying the decreased levels of Ala-substituted mutants of CR1 Ser^612^, Arg^615^, Glu^624^ and Phe^619^ (as well as mutants of the highly conserved CL4-Lys^406^ and CL4/TM8-Asp^430^) remain unknown as our exchange mutagenesis experiments that were guided by both structural and homology based models of MRP1 did not provide any insights. At least at present, a high degree of conservation of an amino acid in CR1 (and CL4) appears to be a better predictor of its structural importance than its potential involvement in interatomic bonding interactions inferred from current static cryo-EM structures and homology models of MRP1. Additional structures with rigorous biochemical validation data are needed to fully understand the bonding interactions critical to the proper folding, assembly and stable expression of this clinically important ABC transporter.

## Supporting information

S1 Fig Alignment of human MRP1 CR1 and CR2 sequences. (TIF)

S2 Fig Effect of bortezomib on levels of poorly expressing MRP1 CR1 mutants S612A, R615A, and E624A in transfected HEK cells. Shown is an immunoblot of extracts (10 μg protein loaded per lane) prepared from HEK cells transfected with wild-type (WT-MRP1) and mutant (S612A, R615A, and E624A) pcDNA expression vectors and then exposed (+) (or not (-)) to bortezomib (100 nM) for 24 h before collecting cells and preparing extracts. Extracts from untransfected cells (HEK) were used as negative controls. Urea (8 M) was included in the protein loading buffer as well as the stacking and resolving gels. The boundary between the stacking and resolving gels is marked with a dashed line. MRP1 was detected with mAb QCRL-1, and anti-Na^+^/K^+^-ATPase was used as a protein loading control. The signals near the top of the blot (indicated by the arrowhead) are aggregates of MRP1.
(TIF)

S1 Table Sequences of MRP1/ABCC1 primers used for mutagenesis in this work. Substituted nucleotides are underlined.
(PDF)

S1 Raw images (PDF)
