# Supplementary figures and images for: Conserved amino acids in the region connecting membrane spanning domain 1 to nucleotide binding domain 1 are essential for expression of the MRP1 (ABCC1) transporter
Source: PLoS One. 2021 Feb 11;16(2):e0246727. doi: 10.1371/journal.pone.0246727 (PMC7877750; doi:10.1371/journal.pone.0246727)

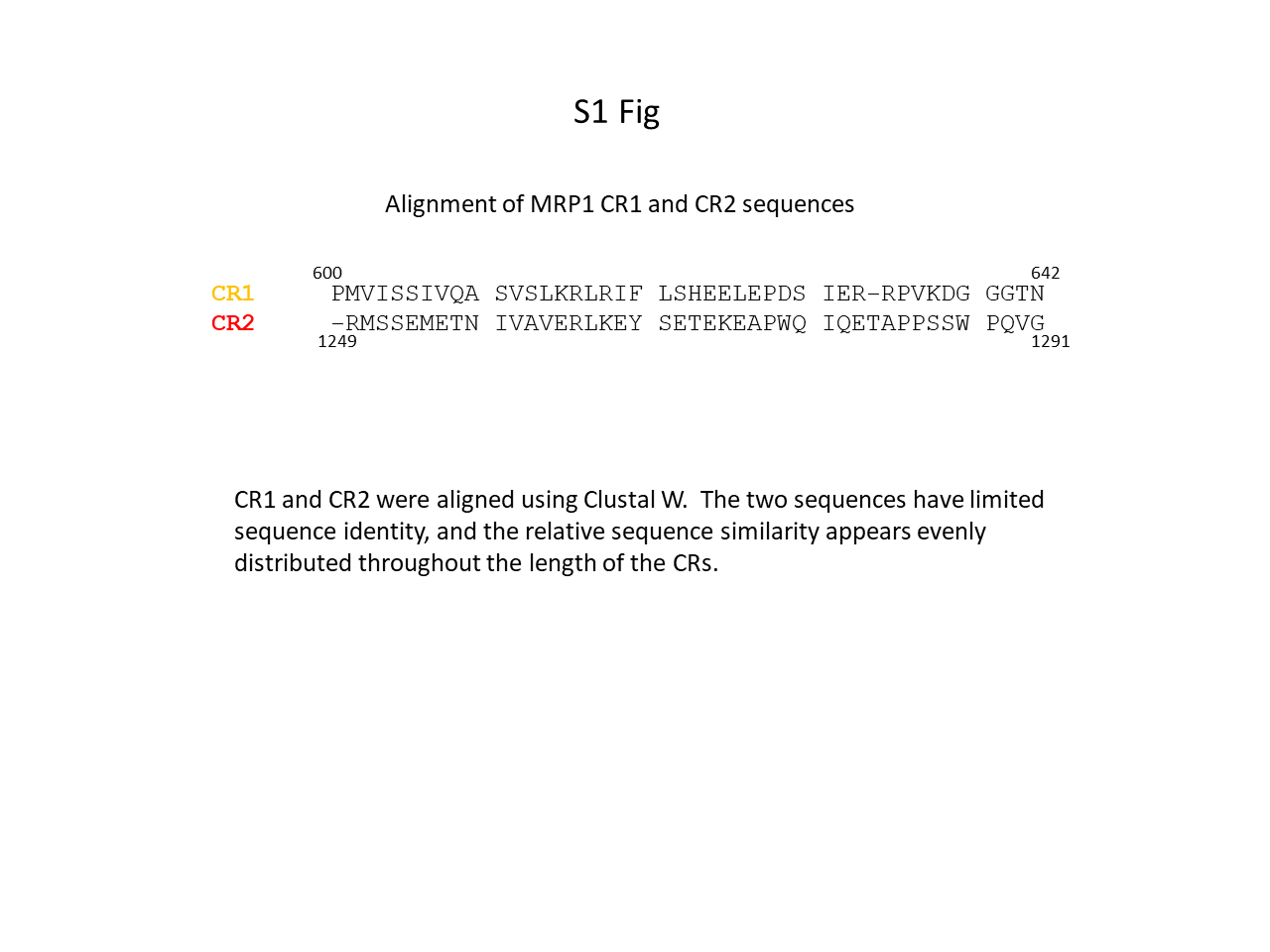

Supplement: S1 Fig — (TIF) [file pone.0246727.s001.tif]

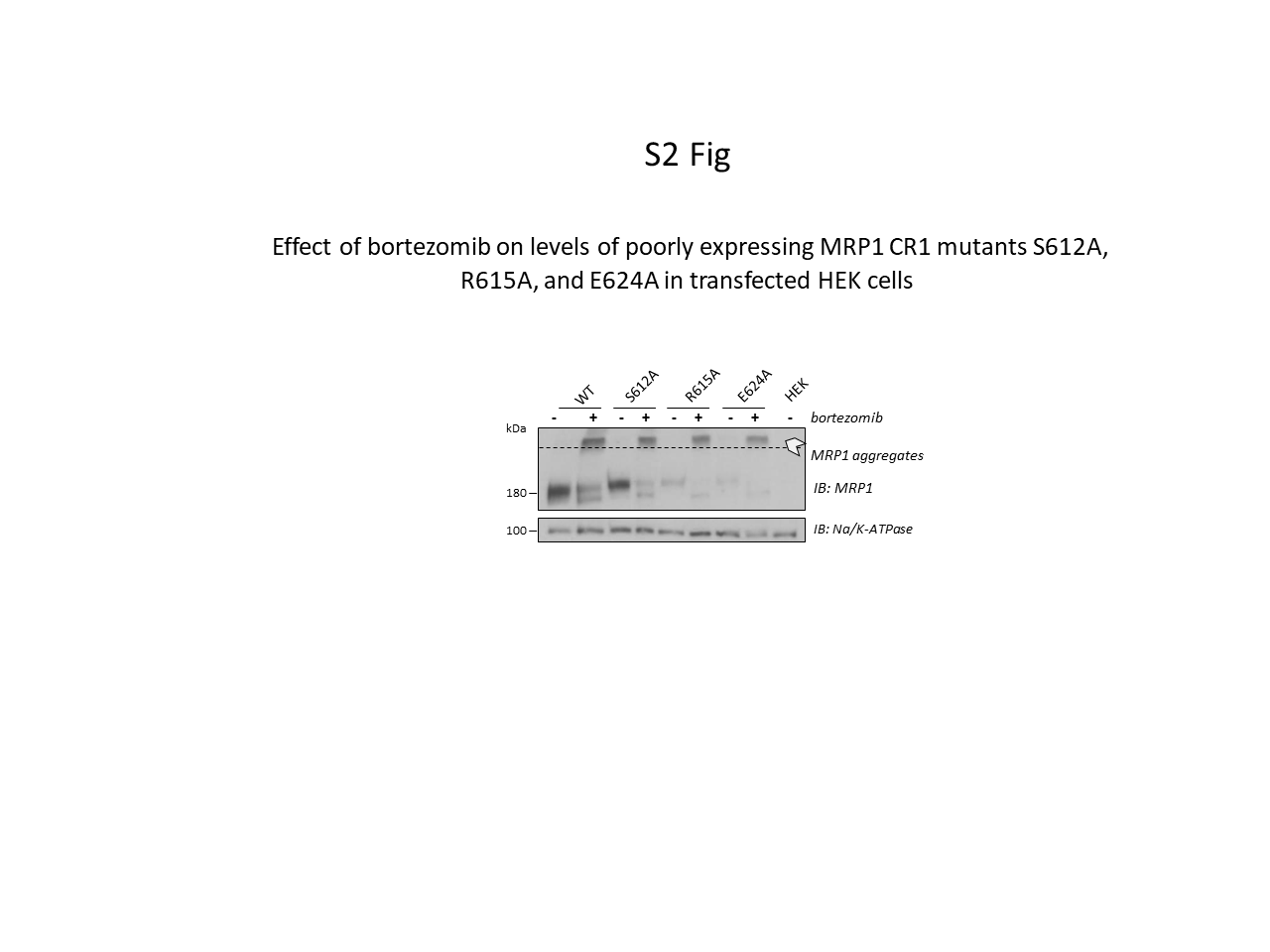

Supplement: S2 Fig — Shown is an immunoblot of extracts (10 μg protein loaded per lane) prepared from HEK cells transfected with wild-type (WT-MRP1) and mutant (S612A, R615A, and E624A) pcDNA expression vectors and then exposed (+) (or not (-)) to bortezomib (100 nM) for 24 h before collecting cells and preparing extracts. Extracts from untransfected cells (HEK) were used as negative controls. Urea (8 M) was included in the protein loading buffer as well as the stacking and resolving gels. The boundary between the stacking and resolving gels is marked with a dashed line. MRP1 was detected with mAb QCRL-1, and anti-Na+/K+-ATPase was used as a protein loading control. The signals near the top of the blot (indicated by the arrowhead) are aggregates of MRP1. (TIF) [file pone.0246727.s002.tif]
