# Supplementary material for: Conserved amino acids in the region connecting membrane spanning domain 1 to nucleotide binding domain 1 are essential for expression of the MRP1 (ABCC1) transporter
Source: PLoS One. 2021 Feb 11;16(2):e0246727. doi: 10.1371/journal.pone.0246727 (PMC7877750; doi:10.1371/journal.pone.0246727)
Supplement: S1 Table — Substituted nucleotides are underlined. (PDF) [file pone.0246727.s003.pdf]

S1 Table. Sequences of MRP1 mutagenesis primers used to create the mutants in this work. Shown are the sequences of the MRP1 mutagenesis primers for site-directed mutagenesis; substituted nucleotides are underlined.

MRP1 Mutant	Sense primer sequence
K406A	5’ CT GTC TAT CGG CCG GCC CTG GTG ATC 3’
K406E	5’ GCT GTC TAT CGG GAG GCC CTG GTG ATC 3’
K406R/K	5’ GCT GTC TAT CGG AAG GCC CTG GTG ATC 3’
D430R	5’ C ATG TCT GTG CGA GCT CAG AGG TTC 3’
S612A	5’ GCG AGT GTC GCC CTC AAA CGC CTG AG 3’
S612A/S	5’ GCG AGT GTC TCC CTC AAA CGC CTG AG 3’
R615A	5’ CG AGT GTC TCC CTC AAA GCC CTG AGG ATC TTT CTC TCC 3’
R615A/R	5’ CG AGT GTC TCC CTC AAA CGC CTG AGG ATC TTT CTC TCC 3’
R615D	5’ GT GTC TCC CTC AAA GAC CTG AGG ATC 3’
R615F	5’ GT GTC TCC CTC AAA TTC CTG AGG ATC TTT CTC TCC 3’
R615K	5’ CG AGT GTC TCC CTC AAA AAG CTG AGG ATC TTT CTC TCC 3’
F619A	5’ C AAA CGC CTG AGG ATC GCC CTC TCC CAT G 3’
F619R	5’ GTC TCC CTC AAA CGC CTG AGG ATC CGG CTC TCC CAT G 3’
F619R/F	5’ CGC CTG AGG ATC TTT CTC TCC CAT GAG GAG C 3’
F619Y	5’ CGC CTG AGG ATC TAT CTC TCC CAT G 3’
R615F/F619R	5’ GTC TCC CTC AAA TTC CTG AGG ATC CGG CTC TCC CAT G 3’
H622A	5’ GC CTG AGG ATC TTT CTC TCC GCT GAG GAG CTG GAA CC 3’
E624A	5’ T CTC TCC CAT GAG GCC CTG GAA CCT GAC AGC 3’
E624A/E	5’ C TCC CAT GAG GAG CTG GAA CCT GAC 3’
E624D	5’ CTC TCC CAT GAG GAC CTG GAA CCT GAC 3’
E624K	5’ C TCC CAT GAG AAG CTG GAA CCT GAC AGC 3’
