# Supplementary material for: Conserved amino acids in the region connecting membrane spanning domain 1 to nucleotide binding domain 1 are essential for expression of the MRP1 (ABCC1) transporter
Source: PLoS One. 2021 Feb 11;16(2):e0246727. doi: 10.1371/journal.pone.0246727 (PMC7877750; doi:10.1371/journal.pone.0246727)

Smith, Conseil & Cole, MRP1 CR1

Uncropped FIGURES – PLoS ONE D-20-28317

Fig 3

A

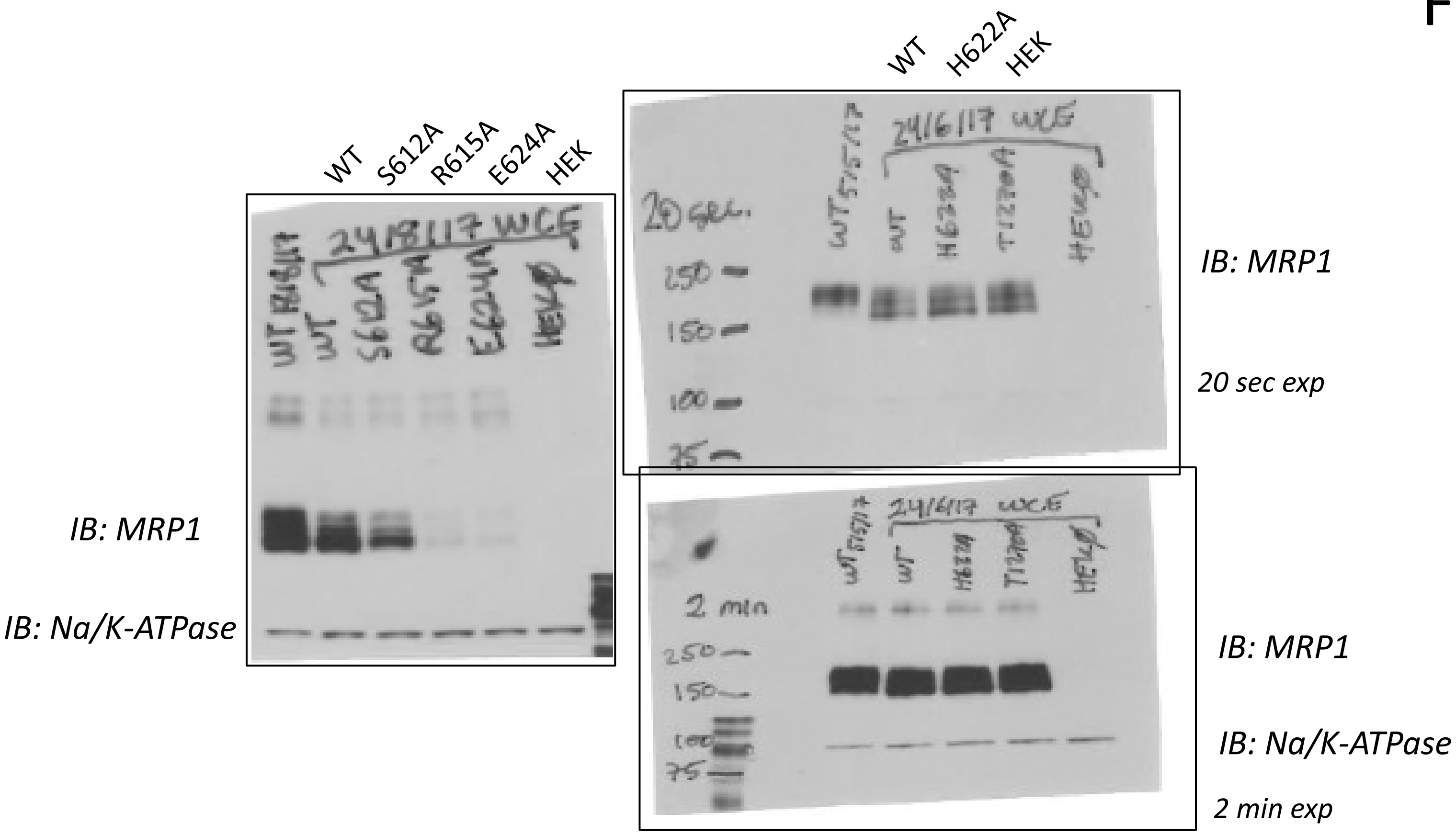
WT
S612A
R615A
E624A
HEK
IB: MRP1
IB: Na/K-ATPase
WT
H622A
HEK
IB: MRP1
20 sec exp
IB: MRP1
IB: Na/K-ATPase
2 min exp

C

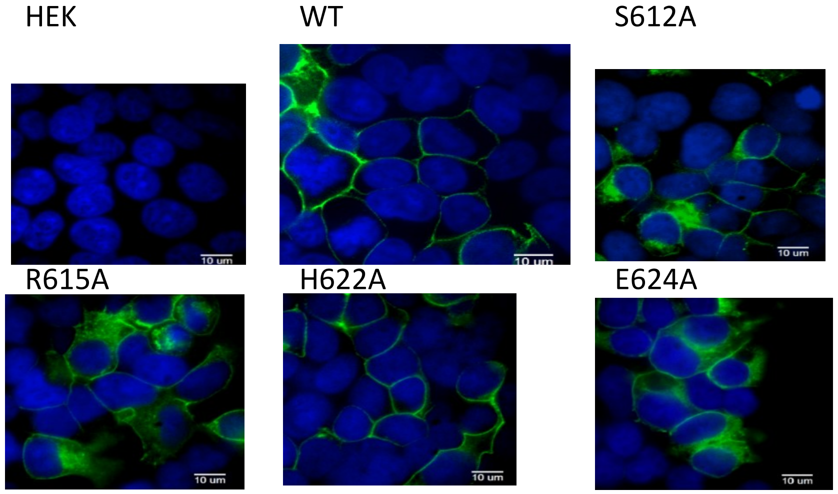
HEK
WT
S612A
R615A
H622A
E624A

Fig 4

A

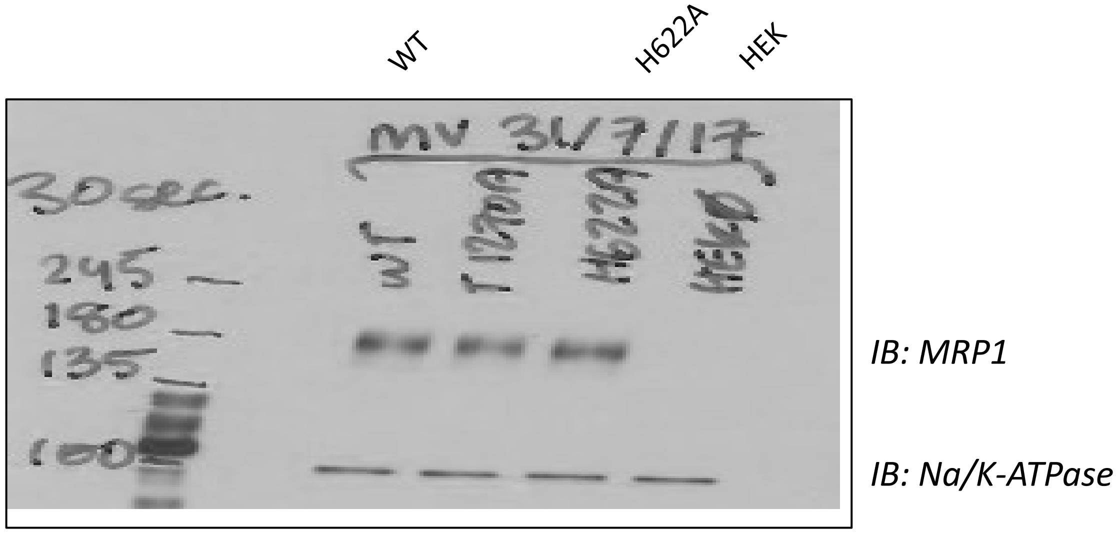

30 sec exp

Fig 5

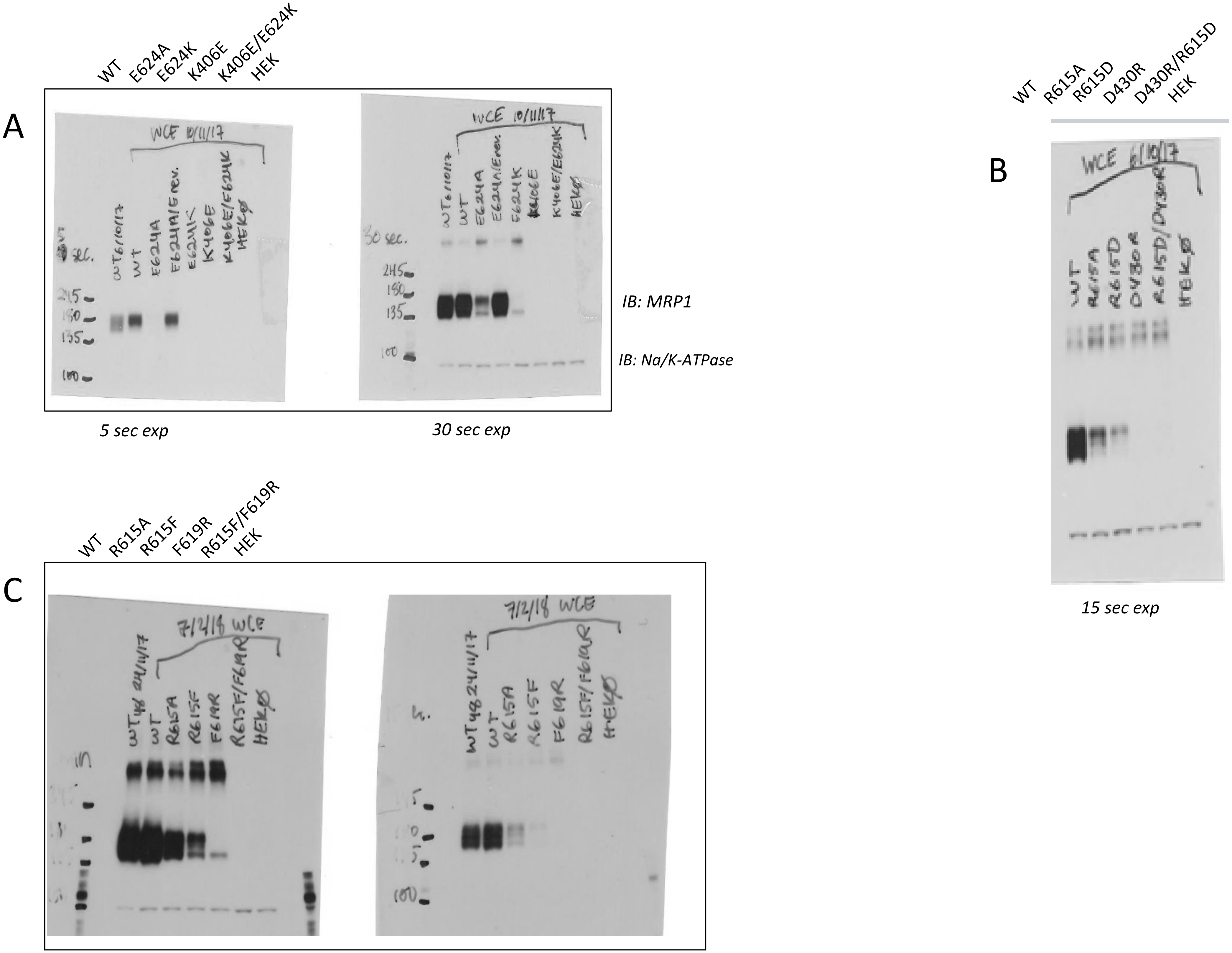

A
WT
E624A
E624K
K406E
K406E/E624K
HEK
IB: MRP1
IB: Na/K-ATPase
5 sec exp
30 sec exp
B
WT
R615A
R615D
D430R
D430R/R615D
HEK
15 sec exp
C
WT
R615A
R615F
F619R
R615F/F619R
HEK

Fig 6

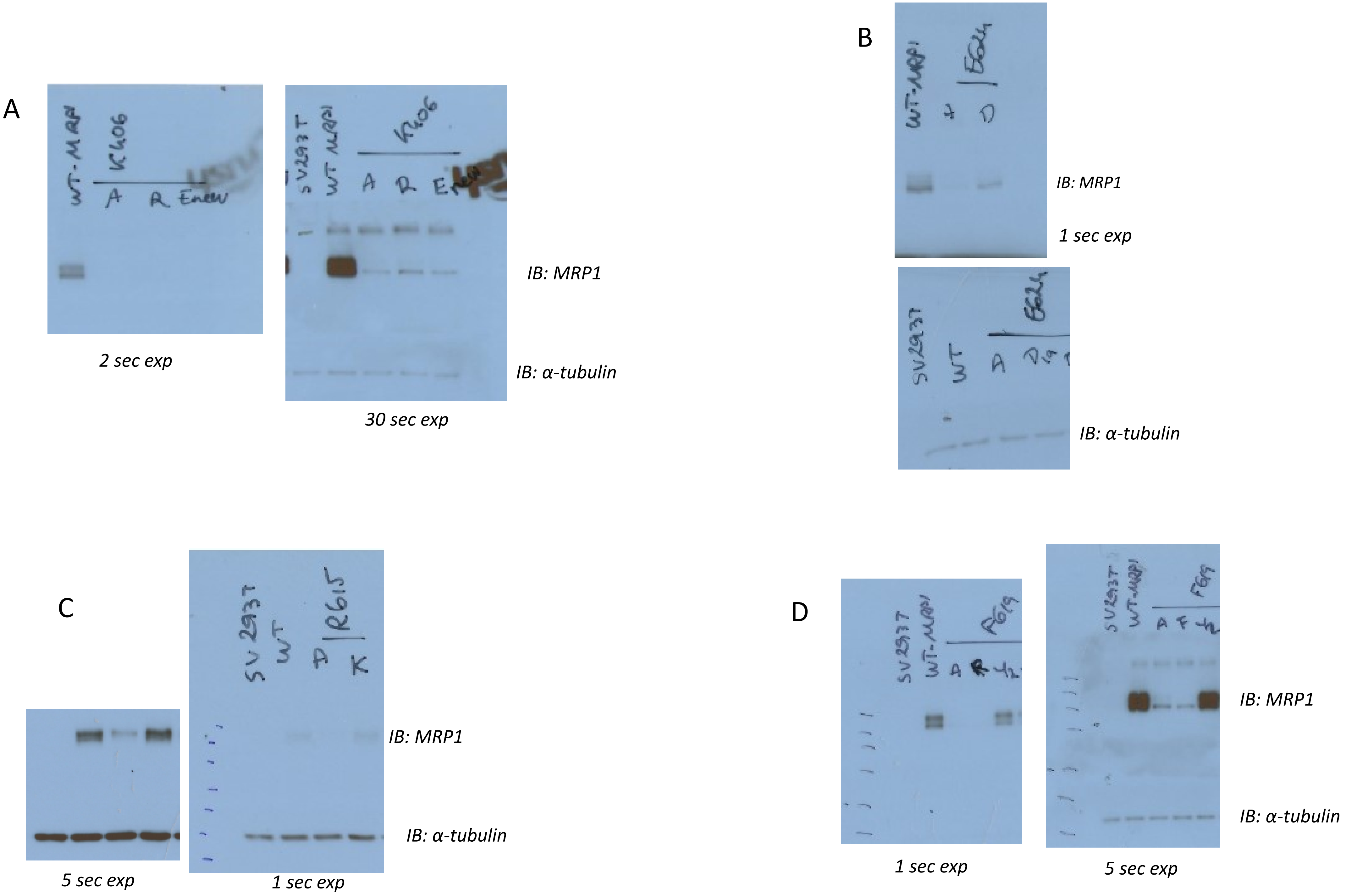
A
IB: MRP1
IB: α-tubulin
2 sec exp
30 sec exp
B
IB: MRP1
1 sec exp
IB: α-tubulin
C
IB: MRP1
IB: α-tubulin
5 sec exp
1 sec exp
D
IB: MRP1
IB: α-tubulin
1 sec exp
5 sec exp

S2 Fig

Effect of bortezomib on levels of poorly expressing MRP1 CR1 mutants S612A, R615A, and E624A in transfected HEK cells

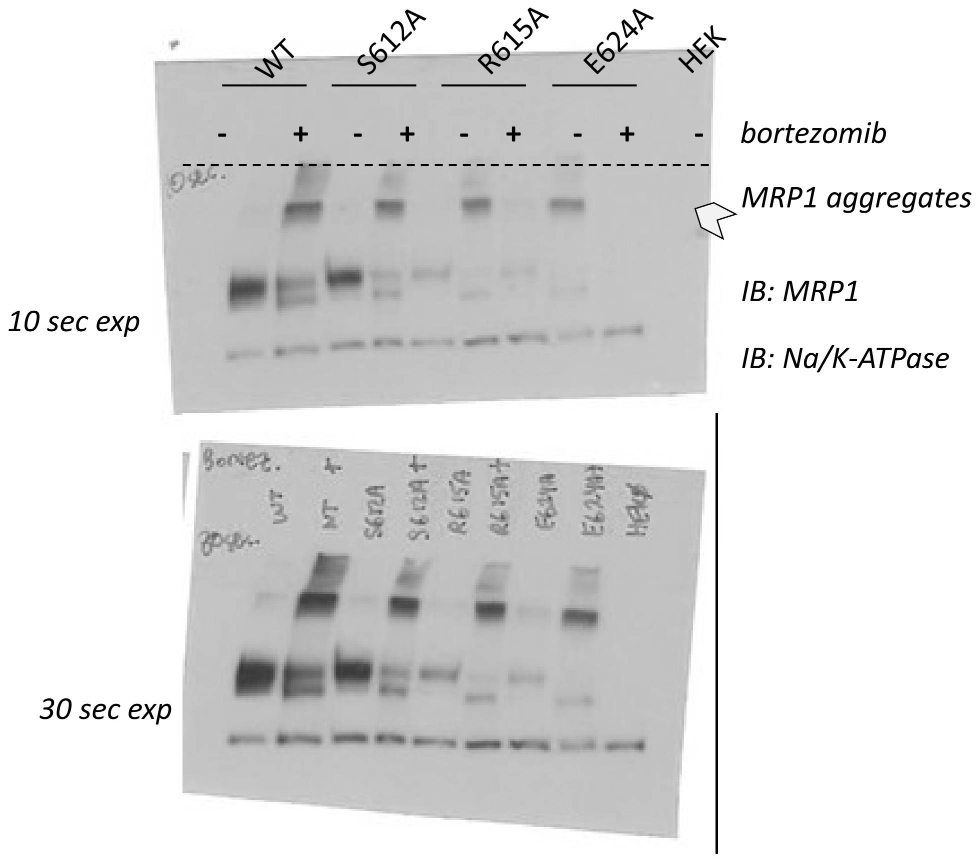

Supplement: S1 Raw images — (PDF) [file pone.0246727.s004.pdf]
